# Efficient and Robust Crystal Plasticity Parameter Identification via RSM-GA Coupling: Application to AZ31 Magnesium Alloy with Bimodal Non-Basal Texture

**DOI:** 10.3390/ma19050919

**Published:** 2026-02-27

**Authors:** Sha Zhan, Li Wang, Jie Sun, Li Hu

**Affiliations:** 1 College of Material Science and Engineering, Chongqing University of Technology, Chongqing 400054, China; 2 Technology Center of Chongqing Gear Box. Co., Ltd., Chongqing 402260, China

**Keywords:** AZ31 magnesium alloy, visco-plastic self-consistent model, parameter identification, genetic algorithm, response surface methodology

## Abstract

**Highlights:**

- RSM-GA framework with a weight-amplified multiscale calibration strategy is developed for efficient crystal plasticity parameter identification.
- Multi-orientation calibration is essential for accurate cross-scenario mechanical and microstructural predictions.
- Material parameters based on the constructed RSM-GA framework effectively capture the plastic deformation behavior and microstructural evolution of AZ31 Mg alloy with bimodal non-basal texture under a variety of loading paths.

**Abstract:**

Identifying material parameters in crystal plasticity constitutive models with high precision and high efficiency can be especially complicated due to the ever-increasing complexity of the models. These material parameters are typically calibrated through the fitting of macroscale experimental data, such as true stress–strain curves, while microscale experimental data, including phase evolution, twinning volume fraction, and so on, are rarely considered and used for verification. In the present study, a novel and computationally efficient optimization procedure for material parameters identification in a crystal plasticity constitutive model has been proposed, which couples a response surface model (RSM) and a genetic algorithm (GA). Specifically, 34 macroscopic true stress–strain data (21 for rolling direction, RD, and 13 for transverse direction, TD) and 4 microscale {10-12} extension twin (ET) volume fraction data have been utilized for multi-objective training. Furthermore, the objective function has been optimized in the present study by tailoring the weights of macroscale stress–strain data and microscale volume fractions for {10-12} ET. The proposed optimization methodology has been verified via visco-plastic self-consistent (VPSC) simulation of tensile deformation for AZ31 magnesium (Mg) alloy sheet with bimodal non-basal texture at room temperature. Results show that the fitness value of the optimization procedure would rapidly converge to a stable value of ~80 within 200 iterations. The obtained material parameters for VPSC simulation on the basis of RD-tensile and TD-tensile experimental data show good validity and applicability in aspects of mechanical response, activities of involved deformation mechanisms, evolution of volume fraction for {10-12} ET, and characteristics of texture evolution.

## 1. Introduction

Owing to the diversity of involved plastic deformation mechanisms in magnesium (Mg) alloys, non-basal slip systems, including prismatic <a> slip and pyramidal <c+a> slip, could also be activated in addition to basal <a> slip during plastic deformation [1,2]. Furthermore, deformation twinning also serves as a crucial mechanism for accommodating plastic strain in Mg alloys, typically through the activation of {10-12} extension twin (ET) and {10-11} compression twin (CT) [3,4]. These finally result in rather complex deformation behavior for Mg alloys. Currently, advanced experimental characterization techniques such as field emission scanning electron microscopy (FESEM), electron backscatter diffraction (EBSD), transmission electron microscopy (TEM), and high-resolution transmission electron microscopy (HRTEM) have been extensively employed in identifying the deformation mechanisms activated during plastic deformation of Mg alloys and qualitatively investigating the interactions among different deformation mechanisms [5,6,7]. However, these experimental characterization methods are inadequate for in situ tracking of microstructure and texture evolution of Mg alloys during plastic deformation, making it difficult to quantitatively assess the contributions of various deformation mechanisms to such evolution. Nowadays, crystal plasticity simulations, which link the grain-level deformation response to that of a polycrystalline aggregate, have become an increasing focus of research about plastic deformation of Mg alloys for determining the physical processes that cause different alloys within a class to exhibit distinct deformation behavior [8,9,10].

Researchers have achieved a consensus that the reliability and accuracy of numerical simulation for plastic deformation of metallic materials via crystal plasticity simulation highly depend on the chosen values for these applied material parameters in constitutive models [11,12,13]. In fact, the identification of material parameters requires solving an inverse problem, which involves adjusting material parameters so that simulation results can match experimental or reference data. The trial-and-error method has been termed as an effective and straightforward approach to determine these material parameters in crystal plasticity constitutive models for copper alloy [14], aluminum alloy [15], NiTi shape memory alloy [16], Mg alloy [11], and so on by means of observing the difference between the predicted stress–strain curves and the experimental ones. However, the proper operation of the trial-and-error method greatly depends on the user’s experience. Furthermore, this approach would become inefficient and even impractical for complex crystal plasticity constitutive models, which involve a large number of material parameters with nonlinear interactions. Andrade-Campos et al. [17] and Herrera-Solaz et al. [18] have reported that the gradient-based optimization methods, including Newton’s method and the steepest descent method, could serve as another effective solution for identifying these material parameters in crystal plasticity constitutive models via the comparison of these measured and predicted tensile curves. However, the convergence of applied gradient-based optimization methods is highly sensitive to the initial estimates of material parameters. This sensitivity poses a significant risk of the algorithm converging to a local optimum, thereby failing to identify the globally optimal solution [17]. Therefore, these reported gradient-based optimization methods are also not appropriate for identifying material parameters in crystal plasticity constitutive models, involving complex deformation mechanisms and strong nonlinear responses.

Kolda et al. [19] and Qu et al. [20] have reported that the abovementioned drawbacks of gradient-based optimization methods for identifying material parameters in the crystal plasticity constitutive model can be effectively tackled by using a direct search method, such as a genetic algorithm (GA). In fact, GA operates through evolutionary processes and crossover recombination mechanisms, offering the advantage of not requiring gradient information from the objective function. However, each iteration of GA requires executing a substantial number of crystal plasticity simulations, followed by the same scale evaluations of the objective functions. This finally results in high computational costs and low efficiency for the GA-based determination of material parameters in crystal plasticity constitutive models, particularly for those with highly complex constitutive relations. More recently, Sedighiani et al. [12] have proposed a computationally efficient and fully automated approach to identify material parameters in crystal plasticity constitutive models from macroscopic tests by searching a response surface using GA and selectively updating the response surface using crystal plasticity simulations. This algorithm integrates the advantages of both response surface model (RSM) and GA, and it could significantly reduce the computational cost associated with crystal plasticity simulations during the material parameter identification process. The merits of this methodology have been verified on examples of rather complex crystal plasticity constitutive models with a large number of material parameters, including a dislocation-density-based crystal plasticity model for Nickel-based superalloy [21] and a phenomenological crystal plasticity model for Mg alloy [12].

It is noteworthy that in references [12,21], only macroscale mechanical data have been applied during the material parameter identification process. This is responsible for the occurrence of non-negligible error with respect to the microscale microstructural characteristics. Savage et al. [22] and Veasna et al. [23] have reported that incorporating more than macroscale mechanical data as objectives is indeed essential in improving the validity and accuracy of material parameter identification for crystal plasticity constitutive models. Therefore, in the present study, a natural extension of the work in references [12,21] has been made for efficiently and robustly verifying material parameters in crystal plasticity constitutive models via collectively fitting the macroscale mechanical responses and microscale microstructural characteristics by using the RSM-GA algorithm. Moreover, considering the limited scale of microscale microstructural characteristics, which can be provided by experimental characterization, a weight amplification technique has been proposed and applied in the present study during the evaluation of the objective functions. To further verify the capability and accuracy of the proposed algorithm, the material parameter of a phenomenological crystal plasticity model incorporating twinning deformation has been identified via visco-plastic self-consistent (VPSC) simulation [24]. The chosen material in the present study is a newly reported AZ31 Mg alloy sheet with bimodal non-basal texture, which is manufactured by using the equal channel angular rolling and continuous bending process with subsequent annealing (ECAR-CB-A) [25]. The rationale for selecting this specific AZ31 Mg alloy sheet lies in its complex microstructural evolution during plastic deformation, which involves not only twinning mechanisms but also dislocation-mediated mechanisms [26].

## 2. Material and Computational Framework

### 2.1. Experimental Process

**Figure 1:**
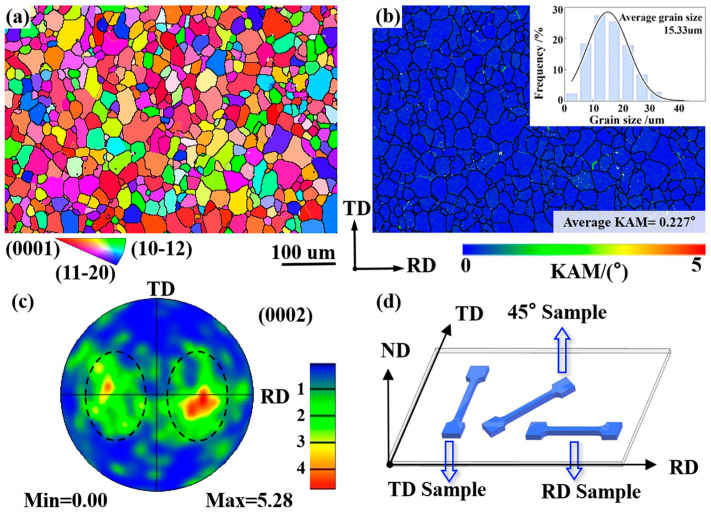
Initial microstructure of applied AZ31 Mg alloy sheet with RD-split bimodal non-basal texture: (**a**) IPF map; (**b**) KAM map inserted with the statistical analysis of grain size; (**c**) (0002) PF showing the tilted basal poles from ND to RD; (**d**) Schematic diagram of sampling for tensile samples.

The applied bimodal non-basal textured AZ31 Mg alloy sheet in the present study was manufactured using the ECAR-CB-A process. The specific procedures and processing conditions had been well documented in reference [25]. Figure 1a–c shows the EBSD measurement results of the fabricated sheet via the inverse pole figure (IPF) map, the kernel average misorientation (KAM) map, and the (0002) pole figure (PF) map. The sample preparation method for EBSD measurement has been reported in reference [27]; therefore, it will not be declared here. Obviously, the applied sheet is mainly composed of equiaxed grains with an average grain size of about 15 μm. As is highlighted by the black dashed oval in Figure 1c, the c-axes of most grains deviate approximately ±40° from the normal direction (ND) toward the rolling direction (RD). These observations agree well with the reports in Chen et al. [26] for a similar RD-split bimodal non-basal textured sheet. Figure 1d shows the sampling strategy for tensile specimens, where the specific dimensions are the same as those reported in [28]. In fact, three kinds of uniaxial loading conditions were applied in the present study, including a load along RD (which is referred to as the RD sample), a load along TD (which is referred to as the TD sample), and a load along 45° to RD (which is referred to as the 45° sample). As the first two loading conditions had already been performed and reported in reference [28] for the studied bimodal non-basal textured AZ31 Mg alloy sheet, only the last loading condition would be conducted in the present study at a constant strain rate of 0.001 s^−1^. To confirm the validity of the obtained mechanical data, tensile experiments were repeated three times. In addition, to compare the measured microscale microstructure characteristics with those predicted ones via VPSC simulation, 45° samples were separately stretched to deformation degrees of 6% and 12%, which is followed by EBSD characterization. The selection of 6% and 12% strain levels aims to represent the characteristic stages of small and large deformation, respectively, considering that the total elongation of the 45° sample is approximately 20%. This dual-stage characterization approach, which has been effectively utilized in previous studies [27,28] to investigate the microstructural evolution of Mg alloys, provides a more comprehensive benchmark to verify the predictive capability of the VPSC model across different deformation regimes.

### 2.2. Optimization Methodology

In the present study, VPSC simulation based on a phenomenological crystal plasticity model has been applied to analyze the plastic deformation behavior of AZ31 Mg alloy sheet with RD-split bimodal non-basal texture. As Lebensohn et al. [29] and Li et al. [30] have provided a detailed account of the VPSC code, only key equations about the hardening models of slip/twinning modes were depicted below.

The extended Voce law proposed by Tomé and Lebensohn, R.A [29,31] was applied in the present study to describe the hardening behaviors of involved slip/twinning modes. This law was selected because it accurately captures the hardening saturation of Mg alloys [31] while maintaining a concise and manageable number of materials parameters for crystal plasticity modeling. Although alternative physics-based models are available, this law remains a standard and efficient choice for VPSC-based texture analysis [32,33,34]. Specifically, it is characterized by an evolution of the critical resolved shear stress (CRSS) ($\tau^{S/\mathrm{TW}}$) with the accumulated shear strain (Γ) in each grain of the form:

(1) $$\tau^{S/\mathrm{TW}}=\tau_{0}^{S/\mathrm{TW}}+(\tau_{1}^{S/\mathrm{TW}}+\theta_{1}^{S/\mathrm{TW}}\Gamma )(1-\exp(-\Gamma \left|\frac{\theta_{0}^{S/\mathrm{TW}}}{\tau_{1}^{S/\mathrm{TW}}}\right|))$$

where $\tau_{0}^{S/\mathrm{TW}}$, $\tau_{1}^{S/\mathrm{TW}}$, $\theta_{0}^{S/\mathrm{TW}}$ and $\theta_{1}^{S/\mathrm{TW}}$ are material parameters, which control the evolution of CRSS ($\tau^{S/\mathrm{TW}}$) for each slip/twinning mode. In the present study, three slip modes, including basal <a> slip, prismatic <a> slip, and pyramidal <c+a> slip, and two twinning modes, including {10-12} ET and {10-11} compression twinning (CT), were considered. This would result in 20 material parameters to be fitted.

To tackle the twinning reorientation issue during crystal plasticity simulation, the predominant twin reorientation (PTR) criterion proposed by Tomé et al. [24] was applied in the present study, which possesses the advantage of keeping invariant the number of grains used to represent the aggregate. The PTR criterion is based on replacing a current orientation (parent) by the twin-related (child) orientation when certain conditions are met, as follows:

(2) $$V^{\mathrm{thres}}=A^{\mathrm{th}1}+A^{\mathrm{th}2}\frac{V^{\mathrm{eff}}}{V^{\mathrm{accum}}}$$

where $V^{thres}$ represents the threshold value of twin volume in each grain, $V^{eff}$ stands for the effective twin fraction and $V^{accum}$ refers to the accumulative twin fraction in a grain. Moreover, $A^{th1}$ and $A^{th2}$ are material parameters, which stand for the lower limit and upper limit of $V^{thres}$, and they control the activation and termination of twin mode during plastic deformation. Considering that {10-12} ET and {10-11} CT were included in the present study, an additional 4 material parameters need to be fitted.

**Figure 2:**
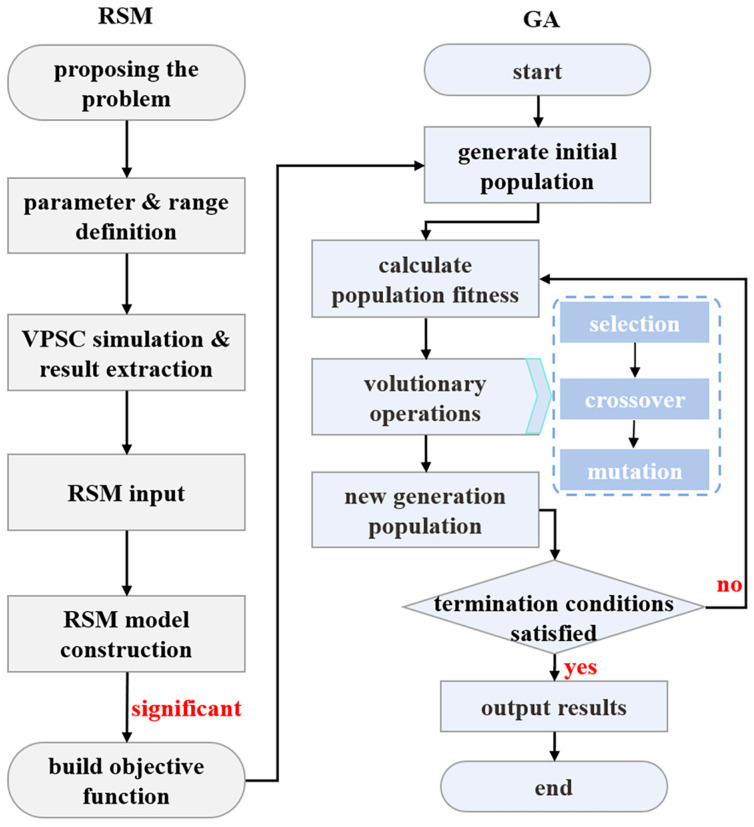
Flowchart of the constructed optimization procedure for material parameters used in VPSC simulation by coupling RSM and GA.

To identify these 24 material parameters in the adopted crystal plastic constitutive model with high precision and high efficiency, the RSM-GA algorithm proposed by Sedighiani et al. [12] was applied in the present study. The corresponding flowchart of the optimization procedure is shown in Figure 2. As the theory and procedures of the RSM-GA algorithm have been well documented in reference [12], only the main process was depicted in the present study.

Actually, the RSM-GA algorithm is a two-stage optimization strategy. In the first stage, a large dataset was generated through performing VPSC simulations by adjusting these material parameters within chosen ranges. Afterwards, an RSM model was constructed using second order polynomial to provide an approximate relationship between independent variables (24 material parameters in the adopted crystal plastic constitutive model) and dependent variables (data about macroscale mechanical responses and data about microscale microstructural characteristics). In the present study, the data about macroscale mechanical responses includes 21 true stress–strain data for RD sample at an interval of plastic strain of 0.01, 13 true stress–strain data for TD sample at an interval of plastic strain of 0.01, and 4 volume fractions for {10-12} ET after deformation degrees of 6% and 12% of both RD sample and TD sample.

The second stage utilized the global search capability of GA to perform parameter optimization based on the sample data generated from this constructed RSM. The objective function was constructed from the significant outcomes of RSM, and it consisted of two parts as specified in the equation below:

(3) ∆E=α+β

where α is designated as the stress error term, and β represents the error term of volume fractions for {10-12} ET. As for the stress error term, it can be further divided into two terms based on the fitting form as follows:

(4) $$\alpha =\sum_{i=1}^{21}\left|x_{i}^{*}-x_{i}\right|+\sum_{i=1}^{13}\left|y_{i}^{*}-y_{i}\right|$$

where $x_{i}^{*}$ and $x_{i}$ represent the experimental and simulated stress values for the RD sample, and $y_{i}^{*}$ and $y_{i}$ represent those for the TD sample at a given plastic strain.

With regard to the error term of volume fractions for {10-12} ET, it can be calculated as follows:

(5) $$\beta =\sum_{k=1}^{2}L(\left|m_{k}^{*}-m_{k}\right|+\left|n_{k}^{*}-n_{k}\right|)$$

where $m_{k}^{*}$ and $m_{k}$ represent the experimental and simulated volume fractions for {10-12} ET of the RD sample at deformation degrees of 6% and 12%, respectively. $n_{k}^{*}$ and $n_{k}$ refer to those for the TD sample at deformation degrees of 6% and 12%, respectively. Moreover, considering that experimental characterization can only provide a limited scale of microscale microstructural characteristics (volume fractions for {10-12} ET), a weight amplification technique has been proposed and applied in the present study during the evaluation of the objective function. This can be realized by adding the term L, which can be obtained by dividing the data about macroscale mechanical responses by the data about microscale microstructural characteristics. In the present study, the weight coefficient L is determined to be 8.5.

Subsequently, the evolutionary process for selecting superior individuals and generating a new population was initiated. The core algorithm can be described by three components:

1. Selection: Simulating natural selection, individuals with high fitness were selected from the i-th generation of the population to be retained in the (i + 1)-th generation of the population. The gene selection probability was set to 0.2 in the present study.
2. Crossover: Simulating sexual reproduction, the selected superior individuals were first randomly paired. For each pair, a portion of their chromosomes was exchanged with a certain probability. The gene crossover probability was set to 0.3 in the present study
3. Mutation: Parameter values were altered through random perturbation within predefined search ranges. A mutation probability of 0.25 was selected based on established literature [12,21] and further refined via a trial-and-error process. Given the complexity of optimizing 24 interdependent parameters, this relatively high rate ensures sufficient genetic diversity and prevents the algorithm from falling into local minima.

By iteratively executing the aforementioned steps, the optimal material parameter combination that satisfies the target error requirements would ultimately be obtained.

## 3. Results and Discussion

### 3.1. Simulation Based on Material Parameters Obtained from RD-Tensile or TD-Tensile Experimental Data (Tensile Curve and Twinning Volume Fraction)

**Figure 3:**
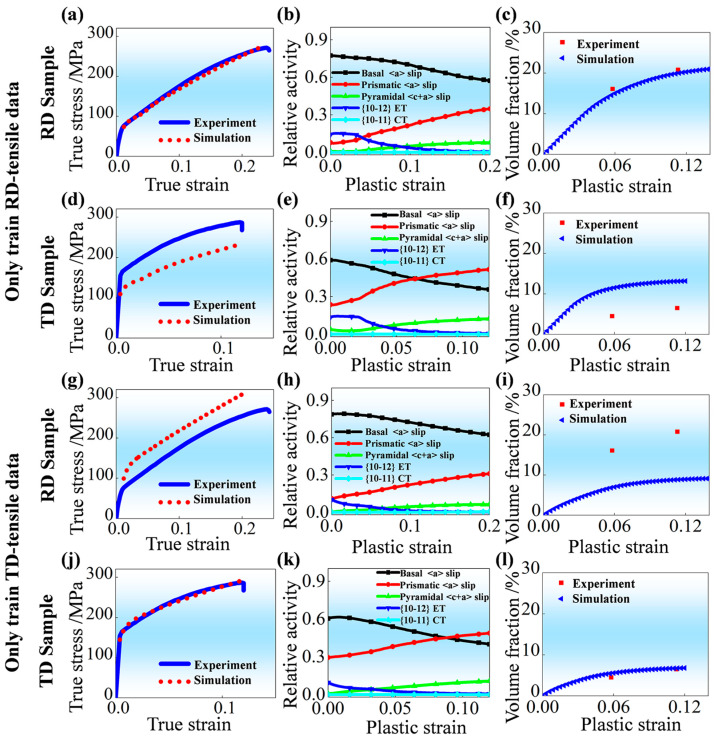
Verification the validity of material parameters for VPSC simulation on basis of RD-tensile or TD-tensile experimental data (tensile curve and twinning volume fraction [28]) by using: (**a**,**d**,**g**,**j**) Comparison between predicted mechanical responses and experimentally measured ones; (**b**,**e**,**h**,**k**) Activities of involved deformation mechanisms; (**c**,**f**,**i**,**l**)Comparison between predicted evolution of volume fraction for {10-12} ET and experimentally measured ones.

Figure 3 displays the VPSC simulation results based on material parameters obtained by training RD-tensile or TD-tensile experimental data (tensile curve and twinning volume fraction). Specifically, it shows the comparison between predicted mechanical responses and experimentally measured ones in Figure 3a,d,g,j, the activities of involved deformation mechanisms in Figure 3b,e,h,k, and the comparison between predicted evolution of volume fraction for {10-12} ET and experimentally measured ones in Figure 3c,f,i,l. The corresponding material parameters are shown in Table 1 and Table 2, respectively. Clearly, by using these fitted material parameters, these predicted results about true stress–strain curves (Figure 3a,j) and volume fractions for {10-12} ET during tensile deformation (Figure 3c,l) are quite close to those experimental results, which are applied for data training. However, the applicability of these obtained material parameters is obviously limited to the corresponding data training scenario. For example, when applying material parameters (Table 1) only on the basis of RD-tensile experimental data (tensile curve and twinning volume fraction), the predicted mechanical response during tensile deformation along TD is notably lower than that obtained from experimental measurement, as shown in Figure 3d. The predicted volume fractions for {10-12} ET in the TD sample are obviously larger than the measured ones, as shown in Figure 3f. The root cause of these significant discrepancies is the strong in-plane anisotropy inherent in AZ31 Mg alloy sheet with RD-split bimodal non-basal texture. In such textured materials, the Schmid factor distributions and the subsequent activities of slip and twinning systems differ dramatically between RD and TD loading conditions [26]. When the optimization is restricted to a single training direction, the genetic algorithm tends to converge to a “local optimum” that over-fits the specific deformation characteristics of that direction. Consequently, these parameters lack the physical universality required to represent the intrinsic properties of the material across different stress states, leading to the failure of cross-validation when the loading axis is changed. This observation strongly demonstrates that it is inappropriate to identify material parameters applied for VPSC simulation only on the basis of RD-tensile or TD-tensile experimental data (tensile curve and twinning volume fraction). It is worth noting that the applied crystal plasticity constitutive model in the present study is only a phenomenological one with few material parameters; those newly developed physics-based models are rather complex and with much more material parameters. Obviously, the operation of identifying material parameters applied for VPSC simulation only on the basis of RD-tensile or TD-tensile experimental data (tensile curve and twinning volume fraction) is also inappropriate for these physics-based models.

In addition, whether comparing Figure 3b,h or comparing Figure 3e,k, it is clear that the most significant difference with respect to the activities of involved deformation mechanisms is the activity of {10-12} ET during plastic deformation. This is responsible for differences with regard to the activities of remaining deformation mechanisms during plastic deformation, as reported by Hu et al. [35]. In fact, the evolution of activity for {10-12} ET is not only associated with the evolution of the current shear stress, which depends on material parameters ($\tau_{0}$, $\tau_{1}$, $\theta_{0}$, $\theta_{1}$), but also closely related to the coefficient associated with twinning reorientation ($A^{th1}$ and $A^{th2}$), which control the activation and termination of twin modes during plastic deformation [11]. From this aspect, it can be concluded that taking the measured microstructure parameter, namely the volume fraction of {10-12} ET, into consideration as training data is quite necessary during the optimization procedure.

### 3.2. Simulation Based on Material Parameters Obtained from RD-Tensile and TD-Tensile Experimental Data (Tensile Curve and Twinning Volume Fraction)

**Figure 4:**
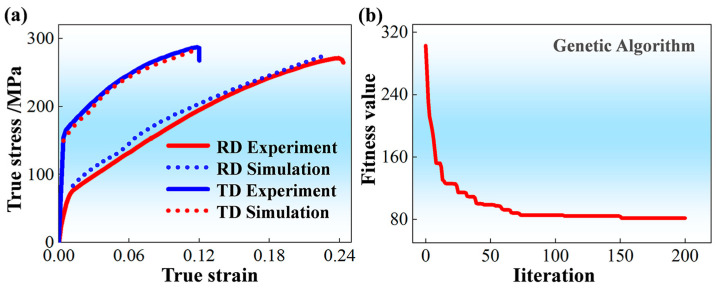
Verification of the validity of material parameters for VPSC simulation on the basis of RD-tensile and TD-tensile experimental data (tensile curve and twinning volume fraction) by using: (**a**) Comparison between predicted mechanical responses and experimentally measured ones; (**b**) Evolution of fitness value during the optimization procedure.

Figure 4a displays the VPSC simulation results about mechanical responses based on material parameters obtained by collectively training the RD-tensile and TD-tensile experimental data (tensile curve and twinning volume fraction). Obviously, by using these obtained material parameters shown in Table 3, VPSC simulation can, in the meantime, predict the corresponding measured true stress–strain curves with high precision. Figure 4b shows the evolution of fitness value with the increasing iteration time. It can be seen that the fitness value of the optimization procedure can sharply reduce to a stable value of ~80 after about 200 iterations. Thereafter, the material parameters in Table 3 can be obtained.

**Figure 5:**
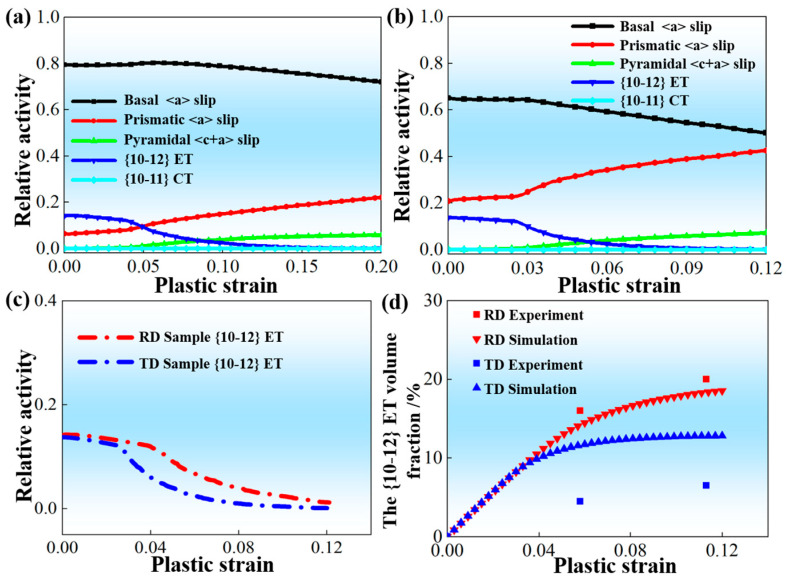
Predicted results of VPSC simulation on basis of RD-tensile and TD-tensile experimental data (tensile curve and twinning volume fraction): (**a**) Activities of involved deformation mechanisms for RD sample; (**b**) Activities of involved deformation mechanisms for TD sample; (**c**) Activities of {10-12} ET; (**d**) Comparison between predicted evolution of volume fraction for {10-12} ET and experimentally measured ones.

Figure 5a,b show the predicted activities of involved deformation mechanisms during plastic deformation by using material parameters on basis of RD-tensile and TD-tensile experimental data (tensile curve and twinning volume fraction). Figure 5c further compares the activities of {10-12} ET during RD-tension and TD-tension. It can be concluded that basal <a> slip always serves as the major deformation mechanism under both loading conditions; meanwhile, {10-12} ET sustains more plastic strain in the RD sample as compared to the TD sample at the early stage of plastic deformation. With the increasing plastic strain, the activity of prismatic <a> slip begins to quickly enhance. However, the corresponding growth rate in the TD sample is obviously larger than that of the RD sample. This phenomenon is in good accordance with the reported results by Hu et al. [35]. Furthermore, whether comparing Figure 5a and Figure 3b for tension along RD or comparing Figure 5b and Figure 3k for tension along TD, it is clear that these predicted activities of involved deformation mechanisms are heavily dependent on these applied material parameters in the VPSC simulation. It is worth noting in Figure 5d that although the predicted evolution of volume fraction for {10-12} ET agrees well with these measured ones during loading along RD, the predicted volume fractions for {10-12} ET in the TD sample are obviously bigger than these measured ones. Kumar et al. [36] and Jin et al. [37] have reported that the activities of involved deformation mechanisms of Mg alloys during VPSC simulation are to sustain plastic strain during plastic deformation, but not to accommodate plastic strain between neighboring grains. In addition, Hu et al. [26] have confirmed that when an AZ31 Mg alloy sheet with RD-split bimodal non-basal texture undergoes tensile deformation along TD, the activation of {10-12} ET rarely occurs, and its role mainly focuses on accommodating local strain between individual grains. Actually, this role of deformation mechanism has not been included in the VPSC simulation at the present form, leading to the overestimation of predicted evolution of volume fraction for {10-12} ET.

**Figure 6:**
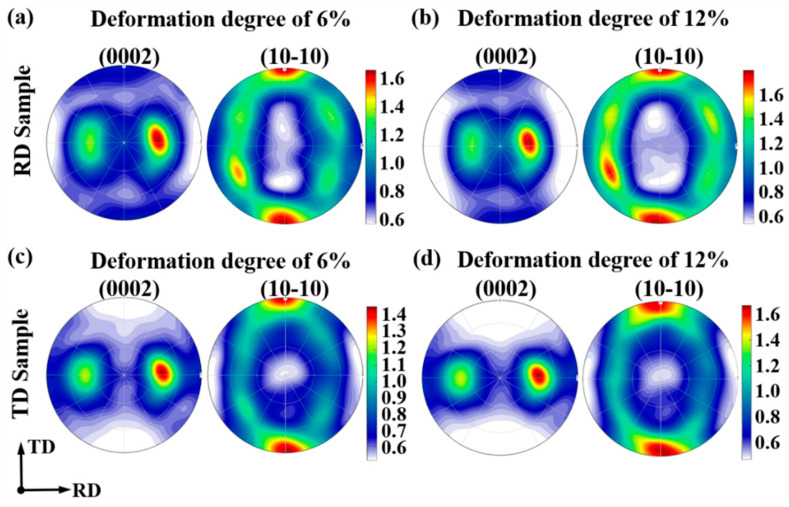
Predicted texture evolution via VPSC simulation on the basis of RD-tensile and TD-tensile experimental data (tensile curve and twinning volume fraction): (**a**,**b**) For RD sample; (**c**,**d**) For TD sample.

Figure 6 shows the predicted texture evolution for the RD sample and the TD sample, respectively, by using the (0002) PF map and the (10-10) PF map. As for the RD sample, the notable characteristic is the concentration of tilted basal poles towards ND in the (0002) PF map during tensile deformation. This phenomenon has also been reported by Chen et al. [27] via EBSD measurement. With regard to the TD sample, the concentration of tilted basal poles towards ND in (0002) PF map is rather weak, indicating the weak activity of {10-12} ET during plastic deformation, as reported by Hu et al. [35].

**Figure 7:**
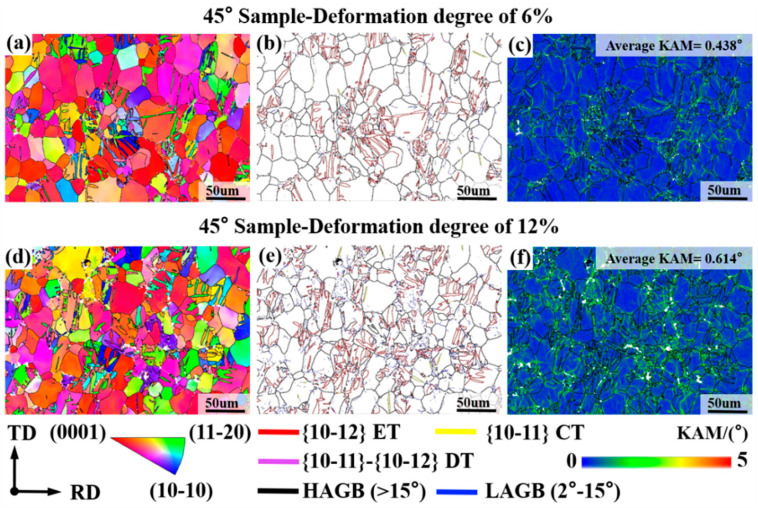
Microstructure characteristics of 45° sample during tensile deformation via EBSD measurement: (**a**,**d**) IPF map; (**b**,**e**) GB map; (**c**,**f**) KAM map.

The abovementioned verifications from the aspects of mechanical response, activities of involved deformation mechanisms, evolution of volume fraction for {10-12} ET, and characteristics of texture evolution have collectively confirmed the validity of the obtained material parameters for VPSC simulation on the basis of RD-tensile and TD-tensile experimental data (tensile curve and twinning volume fraction). To further check the applicability of these obtained material parameters, a 45° sample has been manufactured from AZ31 Mg alloy sheet with RD-split bimodal non-basal texture in the present study, and a tensile experiment has been conducted, as well as microstructure characterization via EBSD measurement. Figure 7 shows the microstructure evolution of the 45° sample during the tensile deformation via an IPF map, GB map, and KAM map. The high density of LAGBs in Figure 7b,e demonstrates that the activation and multiplication of dislocations occurred with high frequency during plastic deformation. This means that dislocation slip plays an important role in plastic deformation to sustain plastic strain in the present study [38]. The KAM results in Figure 7c,f also support this conclusion, where the average KAM value is about 0.438° for the 6%-deformed sheet and about 0.614° for the 12%-deformed sheet, respectively. These values are obviously larger than that (0.227°) of the as-received sheet in Figure 1b. Besides that, it can be seen from Figure 7b,e that there are extensive boundaries of {10-12} ETs highlighted by red lines within deformed samples. This means that {10-12} ET also occurs with high frequency during tensile deformation of the 45° sample. The corresponding volume fractions of {10-12} ET are determined to be about 10.33% for the 6%-deformed sheet and about 14.57% for the 12%-deformed sheet, respectively. By using analysis of twin variants, Hu et al. [26] have confirmed that these activated {10-12} ETs within the deformed 45° sample are mainly used for sustaining plastic strain during tensile deformation.

**Figure 8:**
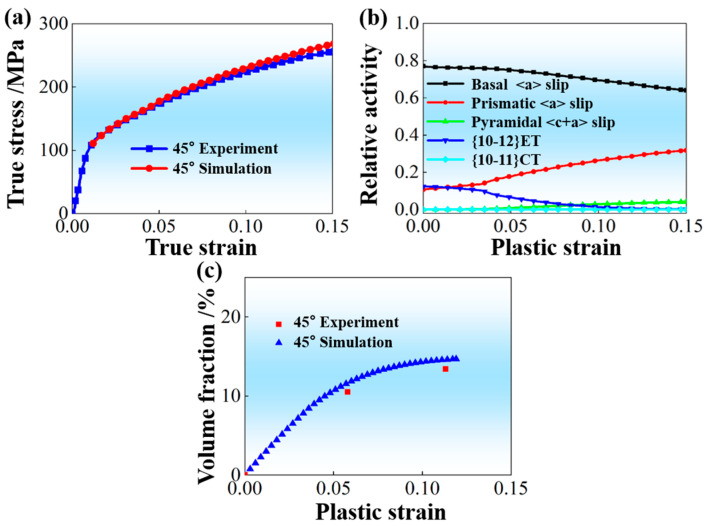
VPSC simulation results of 45° sample by using material parameters on basis of RD-tensile and TD-tensile experimental data (tensile curve and twinning volume fraction): (**a**) Comparison between predicted mechanical response and experimentally measured one; (**b**) Activities of involved deformation mechanisms; (**c**) Comparison between predicted evolution of volume fraction for {10-12} ET and experimentally measured ones.

**Figure 9:**
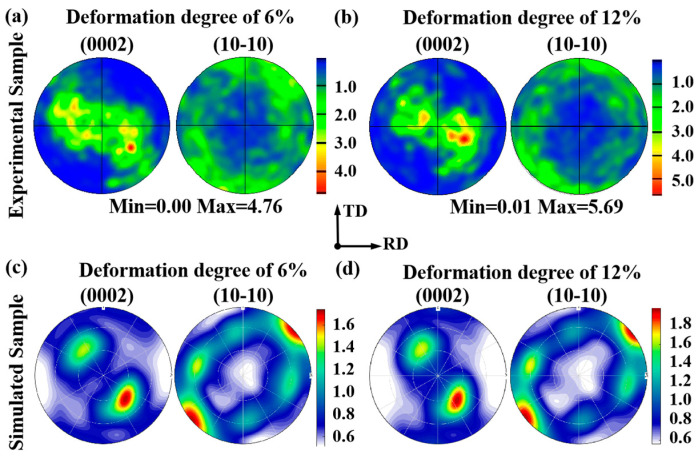
Comparison between experimentally measured texture evolution (**a**,**b**) and the predicted texture evolution (**c**,**d**) of the 45° sample during tensile deformation by using (0002) PF map and (10-10) PF map.

Figure 8 displays the VPSC simulation results of a 45° sample by using material parameters on basis of RD-tensile and TD-tensile experimental data (tensile curve and twinning volume fraction). Both the predicted mechanical response in Figure 8a and the predicted evolution of volume fraction for {10-12} ET in Figure 8c agree well with the corresponding ones by experimental measurement. Specifically, the absolute deviation between the simulated and experimental volume fractions of {10-12} ET is maintained within a narrow range of 1–2%, demonstrating the high fidelity of the microstructural prediction. Meanwhile, the evolution tendency of activities for involved deformation mechanisms in Figure 8b is in good accordance with the previously reported results by Hu et al. [35]. This finally results in an obvious concentration of tilted basal poles towards ND during tensile deformation of the 45° sample, as shown in Figure 9. The abovementioned observations strongly support that the obtained material parameters for VPSC simulation on the basis of RD-tensile and TD-tensile experimental data (tensile curve and twinning volume fraction) possess good applicability to predict the plastic deformation of AZ31 Mg alloy sheet with RD-split bimodal non-basal texture in the present study.

## 4. Conclusions

Identifying material parameters in a crystal plastic constitutive model with high precision and high efficiency has long been a challenging question, which greatly impacts the prediction accuracy of crystal plasticity simulations, including the macroscale mechanical behavior, the evolution of crystallographic texture, the microscale distribution of plastic strain, dislocation density, and so on. To tackle this issue, a novel and computationally efficient optimization procedure for material parameters in the crystal plastic constitutive model has been proposed in the present. The power of this methodology has been demonstrated on the example of a phenomenological constitutive model in VPSC simulation for AZ31 Mg alloy sheet with RD-split bimodal non-basal texture. The following conclusions can be drawn.

(1) The newly constructed optimization approach in the present study has coupled response surface methodology and genetic algorithm. In addition, macroscale experimental data, namely tensile curve, and microscale experimental data, namely twinning volume fraction, have been considered as training data at the same time in order to identify material parameters with high precision and high efficiency. Furthermore, the fitness function, tailored by weighting macroscale stress–strain data and microscale volume fractions, successfully converged to a stable value (~80) after approximately 200 iterations. This process utilized 34 mechanical data points and 4 microscale points, significantly accelerating the identification process while ensuring a reliable error minimization.
(2) Whether it is for a phenomenological constitutive model with few material parameters, or it is for a newly developed physics-based model with many more material parameters, it is inappropriate to identify material parameters applied for VPSC simulation only on the basis of RD-tensile or TD-tensile experimental data (tensile curve and twinning volume fraction). The reason is that the applicability of these obtained material parameters is obviously limited to the corresponding data training scenario. Beyond the data training scenario, the predicted mechanical response and the evolution of volume fraction for {10-12} ET cannot match the experimentally measured ones.
(3) Material parameters for VPSC simulation on the basis of RD-tensile and TD-tensile experimental data (tensile curve and twinning volume fraction) show good validity in predicting mechanical and microstructural evolution. Furthermore, by using these material parameters, the plastic deformation behavior of the 45° sample can be well captured. This shows the good applicability of the proposed optimization procedure, where the deviation between simulated and experimental ET volume fractions is generally within 1–2%.

## Figures and Tables

**Table 1:** Material parameters (MPa) applied for VPSC simulation only on the basis of RD-tensile experimental data (tensile curve and twinning volume fraction).

Deformation Mode	τ_0_	τ_1_	θ_0_	θ_1_	$A^{\mathrm{th}1}$	$A^{\mathrm{th}2}$
Basal <a>	14	10	103	69	-	-
Prismatic <a>	88	18	2639	138	-	-
Pyramidal <c+a>	101	31	3406	150	-	-
{10-12} ET	39	35	83	16	0.610	0.232
{10-11} CT	283	48	406	30	0.087	0.035

**Table 2:** Material parameters (MPa) applied for VPSC simulation only on the basis of TD-tensile experimental data (tensile curve and twinning volume fraction).

Deformation Mode	τ_0_	τ_1_	θ_0_	θ_1_	$A^{\mathrm{th}1}$	$A^{\mathrm{th}2}$
Basal <a>	14	27	115	120	-	-
Prismatic <a>	110	31	2599	150	-	-
Pyramidal <c+a>	155	23	4529	170	-	-
{10-12} ET	75	84	2095	153	0.590	0.202
{10-11} CT	300	53	1328	55	0.054	0.048

**Table 3:** Material parameters (MPa) applied for VPSC simulation collectively on the basis of RD-tensile and TD-tensile experimental data (tensile curve and twinning volume fraction).

Deformation Mode	τ_0_	τ_1_	θ_0_	θ_1_	$A^{\mathrm{th}1}$	$A^{\mathrm{th}2}$
Basal <a>	8.1	45	42	35	-	-
Prismatic <a>	116	49	1311	85	-	-
Pyramidal <c+a>	159	39	4541	66	-	-
{10-12} ET	45	121	200	22	0.736	0.183
{10-11} CT	285	75	1525	43	0.063	0.044

## Data Availability

The raw data supporting the conclusions of this article will be made available by the authors on request.

## Abbreviations

The following abbreviations are used in this manuscript:

CRSS	Critical resolved shear stress
CT	Compression twin
EBSD	Electron backscatter diffraction
ECAR-CB-A	Equal channel angular rolling and continuous bending process with subsequent annealing
ET	Extension twin
FESEM	Field emission scanning electron microscopy
GA	Genetic algorithm
HRTEM	High-resolution transmission electron microscopy
IPF	Inverse pole figur
KAM	Kernel average misorientation
LAGB	Low-angle grain boundary
Mg	Magnesium
ND	Normal direction
PTR	Predominant twin reorientation
RD	Rolling direction
RSM	Response surface methodology
TD	Transverse direction
TEM	Transmission electron microscopy
VPSC	Visco-plastic self-consistent
